# Benign Pediatric Jawbone Lesions: A 10-Year Clinical and Radiological Pilot Study

**DOI:** 10.3390/children10020335

**Published:** 2023-02-09

**Authors:** Emil Crasnean, Alina Ban, Mihaela Hedeșiu, Raluca Roman, Cristian Dinu, Mihaela Băciuț, Sergiu Văcăraș, Ileana Mitre, Oana Almășan, Vlad-I. Nechita, Gabriel Armencea, Simion Bran, Florin Onișor

**Affiliations:** 1 Department of Maxillofacial Surgery and Implantology, Iuliu Hațieganu University of Medicine and Pharmacy, 37 Iuliu Hossu Street, 400029 Cluj-Napoca, Romania; 2 Department of Prosthetic Dentistry and Dental Materials, Iuliu Hațieganu University of Medicine and Pharmacy, 32 Clinicilor Street, 400006 Cluj-Napoca, Romania; 3 Department of Medical Informatics and Biostatistics, Iuliu Hațieganu University of Medicine and Pharmacy, 400349 Cluj-Napoca, Romania

**Keywords:** children, benign, odontogenic tumor, nonodontogenic tumor, odontogenic cyst

## Abstract

This study aimed at presenting a retrospective longitudinal analysis of the pediatric jaw lesions treated at the Oral and Maxillofacial Surgery Clinic in Cluj-Napoca, Romania, over a timeframe of ten years (2012 to 2022). The clinical and radiological characteristics of the jawbone lesions, the treatment outcome, and the recurrence incidence were described. All consecutive patients aged below 18 years, with histologically diagnosed odontogenic tumors (OTs), nonodontogenic tumors (non-OTs), or odontogenic cysts (OCs) were included. Age, dentition type, clinical symptoms, preoperative and postoperative radiological tests, histological diagnosis, treatment, and follow-up information one year following the diagnoses were all examined. Eighty-two cases were included. The ratio of men to women was 1.15:1, with the mandible predominating by 64.4%. Inflammatory radicular cysts predominated in 31.7% of cases. A total of 42.68% of the patients were asymptomatic. Enucleation was the most frequent surgical technique (45.1%), followed by cystectomies (28%) and marsupialization (14.6%). The overall recurrence rate was 7.3%; the most recurrent histopathological lesion was the odontogenic keratocyst. This study sheds new light on the clinical and radiological characteristics, treatment outcomes, and recurrence rate of juvenile jawbone lesions in children and adolescents. The diagnosis and treatment of jawbone lesions in children and adolescents can be enhanced with the use of epidemiological, clinical, and imagistic information.

## 1. Introduction

Pediatric jaw tumors are rare [1] and even though they are generally benign, most lesions can be locally aggressive and can cause functional disorders, cosmetic impairments, and tooth eruption disturbances [2]. The majority of pediatric bone tumors are asymptomatic, being detected incidentally by radiological imaging, whilst possible symptoms are often non-specific: swelling, facial asymmetry, functional disorders, pain, and dental mobility [3]. Any disturbance of the maxillary bone development or asymmetric tooth eruption in children should raise suspicion for a possible jaw lesion and should further be examined radiologically [4].

Pediatric bone tumors encompass a broad variety of pathologic entities. Benign pediatric jaw lesions can arise from odontogenic and non-odontogenic tissues in the maxilla or mandible. The incidence of jaw malignancies is rare in children; however, non-odontogenic benign lesions located at the mandible are the most common pediatric tumors [5].

Radiologically, bone tumors can be classified as radiolucent, radiopaque, or mixed. Some imaging features are specific or even pathognomonic to a certain lesion, but more often the imaging pattern can be encountered in several types of tumors. The radiographic characteristics of bone lesions, such as the tumoral extension, contour, cortical bone expansion, and relationship with the dental roots, play an important role in their differentiation and can be used to extract essential information regarding the aggressiveness of the lesions [6].

To date, the imaging patterns of jaw tumoral conditions have been extensively discussed in adults; nevertheless, there is still a lack of studies reviewing the epidemiological, clinical, and radiological findings in pediatric cases [7]. A potential explanation could be the rarity of pediatric jaw lesions as compared to adult ones and the lower incidence of radiological exposure in children.

Given the heterogeneity of the radiological aspects of jaw lesions, a systematic imaging approach should be considered in the differential diagnosis of this pathology. As orthopantomography (OPG) is the main imaging modality for the evaluation of dental development in children; various jaw lesions can be detected incidentally on panoramic images [8]. Although panoramic radiography could be performed to show differences between bone lesions based on their radiological appearance, this approach allows a relatively limited assessment of the cortical bone and tumoral spreading into the surrounding tissues [9].

Cone beam computed tomography (CBCT) should be always considered a viable alternative to computed tomography (CT) for the evaluation of pediatric bone lesions due to the lower radiation dose [10].

The analysis of the imaging pattern of odontogenic and nonodontogenic lesions in children could have a significant impact on establishing a correct diagnosis rate and appropriate therapeutic management. On the other hand, the term bone tumor encompasses broad entities, such as neoplasms, tumor-like conditions, as well as metabolic diseases [11]. A new international standard for the classification of head and neck tumors and cysts was published in the 5th WHO classification in 2022. The new jaw tumor classification provides many clarifications regarding the defining criteria which can be used in all situations across various global regions [12].

In light of the recently updated classification of tumors, the study of the prevalence of jaw lesions and their radiological features in children could improve differential diagnoses and could contribute to the selection of appropriate surgical treatments. Furthermore, surgical treatments in children should be more conservative compared to adults, given the complex interplay of facial growth and tooth eruption. Adjunctive therapy combined with surgical treatment is also necessary in the case of aggressive lesions. Tumor recurrence is a possibility, and it depends on the pathology and early-stage treatment of tumors [13].

Currently, there are only a few studies that have described/examined the outcomes of surgical treatment in pediatric tumors [3,4,14,15].

This study aimed to provide a retrospective analysis of the pediatric jaw lesions treated at the Oral and Maxillofacial Surgery Clinic in Cluj-Napoca over a ten-year timeframe and to examine their clinical and radiological characteristics, treatment outcomes, and recurrence rate.

## 2. Materials and Methods

A retrospective longitudinal cohort study was performed. All pediatric patients with jaw tumors attending the Department of Maxillofacial Surgery of Cluj-Napoca, Romania were considered eligible.

The inclusion criteria were as follows: all medical records (available imaging and histopathologic exams) of patients with jawbone lesions aged eighteen years or younger, treated between January 2012 and January 2022, were analyzed. The exclusion criteria were as follows: inflammatory pathology and infections, soft tissue and vascular lesions, malignant tumors, salivary gland tumors, and incomplete information, including the lack of radiological imaging or inconclusive histopathologic exam.

All subjects underwent an OPG or a CBCT examination during their presentation. Cases were grouped according to the 2022 WHO classification [12] by the following type of pathologies: odontogenic tumors (OTs), nonodontogenic tumors (non-OTs), and odontogenic cysts (OCs).

For all participants, the following data were collected: age at presentation; dentition type; clinical symptoms; preoperative and postoperative radiological investigations; histopathological diagnosis; therapy; and follow-up data at least 1 year after diagnosis.

The radiological imaging database of patients comprising all available preoperative and examinations was transferred as DICOM files to a workstation at the Department of Oral Radiology and retrospectively analyzed by two board-certified radiologists (M.H. and A.B.). The radiological dataset included the following: lesion location in the jaw, radiographic pattern (radiopaque, radiolucent, mixed), tumor locularity (unilocular, multilocular, pseudolocular), cortical bone changes (expansion or perforation), dental root relationship (resorption, displacement, inclusion, migration), and the involvement of the surrounding structures. The maximum diameter was used to measure the size of the lesions and tumors.

The study obtained ethics approval from the ethical committee of the Faculty of Medicine and Pharmacy “Iuliu Hațieganu”, Cluj-Napoca, Romania (DEP184/26 May 2022).

### Statistical Analysis

The normality of the data was tested using the Shapiro–Wilk test. Continuous variables, such as age and tumor size, were presented as mean and standard deviation. Categorical variables (location of the lesions, locularity, imagistic pattern, symptoms) and dichotomous variables (localization, gender, treatment, recurrence) were presented as numbers and percentages. According to the new WHO classification, benign jaw lesions were divided into 3 major groups: OTs, non-OTs, and OCs. The comparison between the groups was performed using the Chi-square test and, respectively, Fisher’s exact test when expected frequencies were lower than 5. A *t*-test and Mann–Whitney U test were used to compare quantitative values between the groups. To report the results of inferential statistics, the *p*-value was determined, and a *p* < 0.05 significance level of the two-tailed *p*-value for all statistical tests was used. The statistical analysis was performed using the R Commander environment for statistical computing and graphics (R Core Team, Austria, Vienna, 2021) (version 4.0.5).

## 3. Results

From a total number of 121 children and adolescents with sixteen different types of bone lesions who attended the Department of Maxillofacial Surgery during the selected timeframe, eighty-two (44 males and 38 females) children and adolescents who fulfilled the inclusion criteria were selected. There was no statistically significant difference between pediatric males and females in terms of age (*p* = 0.16). The mean age of the participants was 11.23 ± 3.78 years (the age range was from 1 to 18 year). Most of the pediatric patients were in the mixed dentition stage (51.2%) followed by the permanent dentition stage (39%) (Table 1).

Seventeen OTs, fifteen non-OTs, and fifty OCs were identified. The most common osseous lesions were inflammatory radicular cysts (31.7%), followed by odontogenic keratocysts (18.2%) and dentigerous cysts (10.9%). OTs and non-OTs were less common, with a higher prevalence of odontoma (9.76%) and giant cell granulomas (6.1%), while the other jaw tumors had a prevalence of less than 4% (Table 2).

Swelling, pain, and functional disorders related to delayed tooth eruption were the most common symptoms in pediatric tumors. However, most of the patients were asymptomatic (42.68%).

There were no statistically significant differences in terms of the patients’ clinical symptoms at presentation among the different types of tumors studied (Table 3).

### 3.1. Radiological Features

The mandible was the most common location for pediatric jaw lesions (64.6%). The mean jaw lesion size was 2.43 ± 1.4 cm, with a range from 0.5–10 cm and with significant statistical differences between OTs, non-OTs, and OCs (*p* < 0.05). In addition, the majority of the lesions were radiolucent, respectively; all the OCs were radiolucent compared to the OTs and non-OTs (*p* < 0.05). Unilocularity was the most common radiological aspect, followed by multilocularity and pseudolocularity. Significant statistical differences were also found in these three specific categories: the OCs were unilocular, while the radiological aspects of non-OTs and OTs were multilocular or pseudolocular (*p* < 0.05). Root inclusion in the tumor (89.02%) was the most frequent dental change, followed by root displacement and root resorption. Statistically significant results (*p* < 0.05) were also found regarding the extension of the lesions in other anatomical structures, with 86.6% of those in the non-OTs being extensive. However, more than half of the jaw lesions showed cortical expansion and/or perforation (Table 4).

### 3.2. Treatment and Recurrence

The most common surgical procedure was enucleation (45.1%), followed by cystectomy (28%) and marsupialization (14.6%). For subjects with OCs, cystectomy was the main surgical treatment used, and in the case of non-OTs and OTs, enucleation was performed for most of the patients. Postoperatively, 7.3% of the patients underwent bone reconstruction surgery, including iliac crest bone grafts (Table 5).

The overall recurrence rate was 7.3%, with an 11.7% recurrence rate for OTs, 6.6% for non-OTs, and 6% for OCs, respectively, with no statistically significant differences between the groups. Of all the histopathological forms, the most recurrent lesion was the odontogenic keratocyst, followed by ameloblastic fibroma, ameloblastoma, and giant cell granuloma.

## 4. Discussion

Jaw tumors are rare in children and adolescents [16]. The incidence rate is difficult to determine due to the different types of tumors that can be found in pediatric populations [17]. The proportions of pediatric jaw lesions have not always been consistent across previous studies because the prevalence of these lesions can vary depending on the country and the population studied [18].

The present study analyzed eighty-two children and adolescents over 10 years old with 16 different histopathological diagnostics, following the most recent WHO classification. OCs were the most frequently recorded class of lesions (60.9%), followed by OTs (20.7%) and non-OTs (18.2%) (Table 2). Other findings have demonstrated that OCs’ occurrence may differ considerably by demographic region. A study of Das et al. [19] showed a prevalence of 10.7% for OCs. At the same time, a Brazilian investigation revealed that only 12.8% of patients had OCs [20]. These findings demonstrate that the prevalence of these jawbone lesions varies significantly.

Our study has found a 31.7% inflammatory jaw cyst prevalence (Table 2), whereas a different pattern of results (1.5%) was observed by Chen Y et al. [15]. The studies by Daley et al. and Garg et al., which reported a higher prevalence of radicular cysts (65.1%), found a similar pattern of results to those reported in our study [21,22]. The most prevalent type of inflammatory jaw cyst is the radicular cyst, which develops as a result of dental inflammation. The development of these apical cysts is triggered by carious conditions. The identification of OCs is aided by dental radiography or imaging. The well-defined, unilocular radiolucency and well-circumscribed lesions of these cysts can help to identify them. A few occurrences of radicular cysts with a mixed pattern (radiolucent with radiopaque foci) were documented by Sridevi et al. [23]. All of the OCs that we studied had unilocular radiolucency. Radicular cysts have a very limited possibility of causing tooth displacement or root resorption, according to some research [24]. Our research shows that radicular displacement and root resorption occurred in 38% of radicular cysts (Table 4). The results provide evidence that a radicular cyst can have an aggressive nature. Surgical interventions (dental extraction, periapical surgery, and cystectomy), such as endodontic therapy, are one of the treatments in these cases.

The maxillary region is the most frequent site where OCs can be found, followed by the molar region of the mandible. However, according to our research, male children represented the majority of those affected (56%) and the mandible was the most common site for OCs (Table 1).

Our research findings suggest that male dominance and an increased number of mandibular cases were found, in line with the conclusions of Arora et al., 2014 [25]. It has been shown that male subjects exhibit a ratio of 1.6:1 of jaw cysts compared to female subjects [26]. Other studies [27,28] have shown that the maxilla was the most affected bone in pediatric jaw lesions.

Many authors report that the dentigerous cyst and the odontoma are the most frequent OCs and OTs in children, respectively, [29,30,31,32,33,34] which is in agreement with our results. Other researchers have claimed that ameloblastomas (OTs) and fibrous dysplasias (non-OTs) are the most prevalent pediatric jawbone lesions [20,35,36,37]. Contrary to the findings of these studies, our research showed that the radicular cyst was the most common of the OCs, followed by the odontoma of the OTs and the giant cell granuloma of the non-OTs (Table 2). A similar pattern of results was obtained by Siadati et al. [38] in a study spanning 22 years.

The increased rate of developmental cysts indicates that, at the time of a child’s development, factors such as tooth eruption and concomitant jaw growth as well as the likelihood of genetic contributions may interact, resulting in a higher prevalence in the pediatric population [39].

Perry et al. [3] revealed that the majority of pediatric patients were asymptomatic, most of the tumors being incidentally found by OPG. On the contrary, our study showed that the most commonly reported symptoms were swelling and functional disorders (Table 3). This could be linked to the rapid growth of these lesions in the pediatric population, associated with root displacement/resorption and cortical changes, a similar pattern of results to that stated by Zhang et al. [4] and Sharma et al. [40]. Nevertheless, 42.68% of the pediatric patients included in our study were asymptomatic (Table 3). However, in our study, 50% of the OCs also involved other anatomical structures (Table 4). The mandibular canal, nasal fossa, zygomatic bone, or the maxillary sinus were the most common sites for the tumor extension. The results demonstrate that functional problems and swelling were the most prevalent symptoms in our study. These findings highlight the need for imaging in the diagnosis of jawbone lesions.

Imaging exams and interpretation are important tools for determining the aggressiveness of lesions: periosteal reaction, involvement in other structures, cortical bone erosion or perforation, tooth relation (odontogenic/non-odontogenic), and cystic or tissue-like features. Radiographically, the majority of the jawbone lesions were unilocular radiolucencies. Root resorption, tooth migration, and cortical expansion or perforation were uncommon. A similar pattern of results was obtained in 2020 by Chen et al. [15].

The current American Dental Association recommendations are strict and advise against OPG until a child is out of mixed dentition [15]. A radiographic imaging should be performed if there is swelling, a functional issue, or facial asymmetry. When treating a child or adolescent, dental imaging must be as non-invasive as possible [41]. CBCTs have a higher radiation dose compared with conventional radiological methods [42]. Pediatric patients are more vulnerable than adults to the long-term toxicity and carcinogenic consequences of radiation [43,44]. Three-dimensional imaging (3D) displays important details in jawbone tumoral extensions. The majority of our patients had a CBCT or an OPG at their initial appointment. For youngsters and teenagers who exhibit a positive sign in a tumoral lesion, we advise panoramic radiography. A CBCT is among the preferred options for imaging evaluations if the lesion is extensive.

The planning and implementation of treatments of jawbone lesions in pediatric patients represent one of the most difficult challenges of clinical practice since most cysts and tumors tend to recur with conservative management. Multidisciplinary treatments and diagnostics in children and adolescents need an immediate participation between pedodontics, maxillofacial surgeons, ENT doctors, dentists, radiologists, medical oncologists, radiotherapists, and pathologists. In our study, the odontogenic keratocyst was the most frequent type that relapsed due to decompression instead of radical enucleation. Choosing the best surgical treatment for children and adolescents can be one of the most difficult stages. The treatment of benign tumors is usually minimally invasive and for small forms does not require complex bone reconstruction. If surgical resection is necessary for the large benign lesions, it is mandatory to preserve the condylar and subcondylar growth centers. However, radical and more aggressive interventions (osteotomy, lesion resection, chemical agents, such as Carnoy’s solution, cryotherapy, or peripheral osteotomy) can adversely affect the functional, social, and psychological life of the child. Overall, the treatment generally recommended for these pediatric bone lesions ranges from more conservative measures, including marsupialization, to extensive procedures, such as enucleation. In addition, long-term follow-up is obligatory in children’s jaw pathology [3].

In our study, enucleation was the most common type of surgical procedure, being reported in 45.1% of patients (Table 5), which is in line with the findings of Chen et al. [15].

In cases with an inflammatory origin, a cystectomy was the preferred treatment of choice compared to marsupialization because of the small size of the lesions and the ease of curettage. However, superior results were described for large-sized developmental OCs when marsupialization was performed.

The recurrence rate differs, ranging from 0% to 62% [3,45], and was 7.3% in our study. Of all the histopathological forms analysed in our study, the most recurrent lesion was the odontogenic keratocyst. Some studies have shown that the recurrence rate is lower when marsupialization or adjunctive antiangiogenic therapy is used as a method of treatment for developmental cysts [46,47]. In our study, the lowest recurrence rate was for the giant cell granuloma. In some studies, giant cell granulomas were categorized as “hybrid lesions” when they coexisted with other benign tumors and lesions, such as ossifying fibromas and odontogenic fibromas [48]. The “hybrid” aspect is composed of the combined histological and imaging components of the two lesions and co-exists in the same location as other pediatric jaw lesions. This type of jaw lesion was not observed in our study, but it is a significant benign osseous tumor with an important radiological and clinical aggressiveness. Understanding the clinical and radiological relationships between the various types of pediatric jawbone lesions may allow practitioners to diagnose and treat these rare pathologies.

### Study Limitations, Strenghts and Future Directions

The findings of our study, which examined benign jawbone lesions in children and adolescents at the Department of Maxillofacial Surgery in Cluj-Napoca, Romania over a ten-year period, may differ from those of other studies from other specialized hospitals or centers throughout the world. Furthermore, it must be acknowledged that to confirm these findings, a larger patient population or multicenter research is recommended. A multidisciplinary medical team’s experience and knowledge are used in this original study treatment. The follow-up information is an essential direction for future research. Following surgical therapies, imaging studies and clinical evaluations are required. The effects of the face growth could include asymmetry or functional disorders. To prevent any occlusal or temporomandibular alterations following surgical treatment, a multidisciplinary follow-up analysis is required. To emphasize the impact of pediatric benign jaw tumors on the development of the craniofacial skeleton, new research should be emphasized. 

## 5. Conclusions

Pediatric jawbone lesions are rare and typically asymptomatic; they are frequently found by chance during routine dental radiography. The majority of our patients developed jawbone inflammation lesions. Swelling and pain are warning signs in the diagnosis of this pathology. To identify this pathology in its earliest stages, practitioners need to be aware of these symptoms. The results of this study contribute to a better understanding of the prevalence, diagnosis, and treatment of benign pediatric jawbone lesions by revealing details on the lesions’ radiological and clinical characteristics, as well as the effectiveness of treatment and likelihood of recurrence. Epidemiological, clinical, and imagistic characteristics are valuable tools for guiding the treatment of jawbone lesions in children and adolescents.

## Figures and Tables

**Table 1:** Age and gender distribution and dentition type in children and adolescents (*n* = 82).

Variable	Total *n* = 82	OTs *n* = 17	Non-OTs *n* = 15	OCs *n* = 50	*p*-Value
**Gender**					0.16
Male	44 (53.6)	8 (47)	8 (53.3)	28 (56)	
Female	38 (46.3)	9 (52.9)	7 (46.6)	22 (44)	
**Age**					0.38
Mean	11.23 ± 3.78	10 ± 4.24	11 ± 3.18	13 ± 3.19	
Range	1–18	1–13	3–14	4–18	
**Dentition Type**					0.61
Temporary	8 (9.7)	3 (17.6)	2 (13.3)	3 (6)	
Mixed	42 (51.2)	11 (64.7)	10 (66.6)	21 (42)	
Permanent	32 (39)	3 (17.6)	3 (20)	26 (52)	

Data presented as *n* (%) or mean ± standard deviation. Abbreviations: OTs (odontogenic tumors); non-OTs (nonodontogenic tumors); OCs (odontogenic cysts).

**Table 2:** Distribution and prevalence of pediatric jaw lesions according to the 5th WHO classification.

Jaw Lesion	*n* * (%)
**Benign odontogenic tumors (OTs)**	**17 (20.7)**
*Epithelial*	
Ameloblastoma	2 (2.4)
*Mesenchymal*	
Odontogenic fibroma	1 (1.2)
Odontogenic myxoma	5 (6.1)
*Mixed*	
Ameloblastic fibroma	1 (1.2)
Odontoma	8 (9.76)
**Benign nonodontogenic tumors (non-OTs)**	**15 (18.2)**
*Maxillofacial bone tumors*	
Osteoma	1 (1.2)
Osteoid osteoma	1 (1.2)
Osteoblastoma	1 (1.2)
Desmoplastic fibroma	1 (1.2)
*Fibro-osseous tumors*	
Fibrous dysplasia	3 (3.6)
*Giant cell lesions and bone cysts*	
Giant cell granuloma	5 (6.1)
Simple bone cyst	2 (2.4)
Cherubism	1 (1.2)
**Odontogenic cysts (OCs)**	**50 (60.9)**
*Inflammatory*	
Radicular cyst	26 (31.7)
*Developmental*	
Dentigerous cyst	9 (10.9)
Odontogenic keratocysts	15 (18.2)

* Presented as absolute values and percentages; Abbreviations: OTs (odontogenic tumors); non-OTs (nonodontogenic tumors); OCs (odontogenic cysts).

**Table 3:** Symptoms in pediatric jawbone lesions.

Symptoms	Total (*n* = 82)	OTs (*n* = 17)	Non-OTs (*n* = 15)	OCs (*n* = 50)	*p*-Value
Asymptomatic	35 (42.68)	9 (52.94)	4 (26.66)	22 (44)	0.31
Swelling	45 (54.87)	8 (47.05)	11 (73.33)	26 (52)	0.26
Pain	28 (34.14)	5 (29.41)	3 (0.2)	20 (40)	0.32
Delayed Tooth eruption	6 (7.31)	2 (11.76)	2 (13.33)	2 (4)	0.34
Functional Disorders	40 (48.78)	8 (47.05)	9 (0.6)	23 (46)	0.62

Data are presented as absolute values and percentages. Abbreviations: OTs (odontogenic tumors); non-OTs (nonodontogenic tumors); OCs (odontogenic cysts).

**Table 4:** Imagistic and radiographic analysis of jawbone lesions.

Variable	Total (*n* = 82)	OTs (*n* = 17)	Non-OTs (*n* = 15)	OCs (*n* = 50)	*p*-Value
*Size (cm)*	2.43 ± 1.4	2.82 ± 1.01	3.6 ± 2.32	1.96 ± 0.82	<0.01
*Location*					0.67
Mandible	53 (64.63%)	10 (58.82%)	9 (60%)	34 (68)	
Maxillary	29 (35.36%)	7 (41.17%)	6 (40%)	16 (32)	
*Pattern*					<0.01
Radiolucent	70 (85.36%)	9 (52.95%)	11 (73.33%)	50 (100)	
Radiopaque	9 (10.97%)	8 (47.05%)	1 (6.66%)	0	
Mixed	3 (3.6%)	0 (0)	3 (20%)	0	
*Locularity* *					<0.01
Unilocular	54 (77.14)	2 (22.22)	3 (27.27)	49 (98)	
Multilocular	13 (18.57)	7 (77.77)	6 (54.54)	0	
Pseudolocular	3 (4.28)	0 (0)	2 (18.18)	1 (2)	
*Cortical expansion or perforation*	48 (58.53)	11 (64.7)	10 (66.66)	27 (54)	0.47
*Root displacement*	38 (46.34)	11 (64.7)	8 (53.33)	19 (38)	0.54
*Root resorption*	37 (45.12)	9 (52.94)	9 (60)	19 (38)	0.20
*Root inclusion*	73 (89.02)	14 (82.35)	15 (100)	44 (88)	0.13
*Other structures* **	48 (58.53)	10 (58.82)	13 (86.66)	25 (50)	<0.01

Data presented as *n* (%) or median and range. Abbreviations: OTs (odontogenic tumors); non-OTs (nonodontogenic tumors); OCs (odontogenic cysts). * For locularity, only the radiolucent lesions were considered. ** For other structures, mandibular canal involvement, nasal fossa, zygomatic bone and floor of the maxillary sinus were included.

**Table 5:** Treatment of pediatric jawbone lesions.

Treatment	Total (*n* = 82)	OTs (*n* = 17)	Non-OTs (*n* = 15)	OCs (*n* = 50)
*Simply Biopsy*	4 (4.8)	1 (5.8)	3 (2)	0 (0)
*Cystectomy*	23 (28)	0 (0)	0 (0)	23 (46)
*Cystectomy and reconstruction*	1 (1.2)	0 (0)	0 (0)	1 (2)
*Enucleation*	37 (45.1)	12 (70.5)	10 (66.6)	15 (30)
*Enucleation and reconstruction*	5 (6)	2 (11.7)	1 (6.6)	2 (4)
*Marsupialization*	12 (14.6)	2 (11.7)	1 (6.6)	9 (18)

Presented as absolute values and percentages.

## Data Availability

Data are provided within the paper.
